# The 17‐gene stemness score associates with relapse risk and long‐term outcomes following allogeneic haematopoietic cell transplantation in acute myeloid leukaemia

**DOI:** 10.1002/jha2.466

**Published:** 2022-05-23

**Authors:** Dennis D. H. Kim, Igor Novitzky Basso, Taehyung Simon Kim, Seong Yoon Yi, Kyoung Ha Kim, Tracy Murphy, Steven Chan, Mark Minden, Ivan Pasic, Wilson Lam, Arjun Law, Fotios V. Michelis, Armin Gerbitz, Auro Viswabandya, Jeffrey Lipton, Rajat Kumar, Stanley W. K. Ng, Tracy Stockley, Tong Zhang, Ian King, Jonas Mattsson, Jean C. Y. Wang

**Affiliations:** ^1^ Department of Medical Oncology and Hematology Princess Margaret Cancer Centre University of Toronto Toronto Canada; ^2^ Faculty of Medicine University of Toronto Toronto Canada; ^3^ Department of Computer Science University of Toronto Toronto Canada; ^4^ The Donnelly Centre for Cellular and Biomolecular Research University of Toronto Toronto Canada; ^5^ Department of Internal Medicine Inje University Ilsan Paik Hospital Goyang Korea; ^6^ Department of Internal Medicine College of Medicine Seoul Hospital Soonchunhyang University Seoul Korea; ^7^ Cancer Genome Project Wellcome Sanger Institute Hinxton UK; ^8^ The Advanced Molecular Diagnostics Lab Princess Margaret Cancer Centre Toronto Canada; ^9^ Clinical Laboratory Genetics Laboratory Medicine Program University Health Network Toronto Canada; ^10^ Department of Laboratory Medicine and Pathobiology University of Toronto Toronto Canada; ^11^ Gloria and Seymour Epstein Chair in Cell Therapy and Transplantation Princess Margaret Cancer Centre Toronto Canada; ^12^ Department of Medical Oncology and Hematology Princess Margaret Cancer Centre University of Toronto Toronto Canada

**Keywords:** Acute leukaemia, AML, Gene expression, LSC 17 score, Stem cell transplantation

## Abstract

A 17‐gene stemness (LSC17) score determines risk in acute myeloid leukaemia patients treated with standard chemotherapy regimens. The present study further analysed the impact of the LSC17 score at diagnosis on outcomes following allogeneic haematopoietic cell transplantation (HCT). Out of 452 patients with available LSC17 score, 123 patients received allogeneic HCT. Transplant outcomes, including overall (OS), leukaemia‐free survival (LFS), relapse incidence (RI) and non‐relapse mortality (NRM), were compared according to the LSC17 scored group. The patients with a low LSC17 score had higher OS (56.2%) and LFS (54.4%) at 2 years compared to patients with high LSC17 score (47.2%, *p* = 0.0237 for OS and 46.0%, *p* = 0.0181 for LFS). The low LSC17 score group also had a lower relapse rate at 2 years (12.7%) compared to 25.3% in the high LSC17 score group (*p* = 0.017), but no difference in NRM (*p* = 0.674). Worse outcomes in the high LSC17 score group for OS, LFS and relapse were consistently observed across all stratified sub‐groups. The use of more intensive conditioning did not improve outcomes for either group. In contrast, chronic graft‐versus‐host‐disease was associated with more favourable outcomes in both groups. The 17‐gene stemness score is highly prognostic for survival and relapse risk following allogeneic HCT.

## 1 INTRODUCTION

Allogeneic haematopoietic cell transplantation (HCT) is regarded as the only curative treatment modality for acute myeloid leukaemia (AML), through a significant reduction of relapse risk [1]. However, a significant proportion of patients still experience relapse with about a 15% incidence rate at 2 years after HCT [2]. Even higher risk of relapse and worse leukaemia‐free survival (LFS) are observed in AML patients who have undergone HCT in second remission (CR2) or beyond compared to those who underwent HCT in first remission (CR1) [3]. Hence, there is an urgent need for tools to identify patients at high risk of relapse prior to HCT and to inform on the optimal post‐transplant management in patients at high risk of relapse.

AML is a disease entity consisting of heterogeneous sub‐types with diverse pathogenesis pathways and variable prognoses [4, 5]. Disease factors, including cytogenetic abnormalities [6] and molecular profiles [7, 8, 9], remain the strongest indicators for relapse, and stratify patients into favourable, intermediate and adverse risk groups, upon which decisions for consolidation treatment are based [7]. The European LeukaemiaNet [7] has recommended a standardized reporting system of AML classification that incorporates both cytogenetic and molecular genetic profiling and has been widely adopted to identify candidates for allogeneic HCT [10]. Patients classified into intermediate and adverse risk groups are offered allogeneic HCT, whereas HCT is deferred in patients with favourable risk [7]. However, disease factors still affect long‐term outcomes following HCT, as those with adverse cytogenetic risk are expected to have inferior transplant outcomes with a higher risk of relapse compared to those with intermediate risk disease. Additionally, outcomes for patients within risk groups are heterogeneous, particularly for the intermediate risk group. Thus, further tuning of the current risk classification system is needed to aid in management decisions.

A 17‐gene stemness score (LSC17 score) has been reported to determine risk and long‐term outcomes in AML patients treated with standard chemotherapy [11]. A high LSC17 score was robustly associated with increased relapse risk and shorter survival in five independent AML validation cohorts (total n = 908). Allogeneic HCT did not improve overall survival (OS) compared to a non‐transplant approach for either high (*p* = 0.2) or low (*p* = 0.06) LSC17 score patients, possibly due to competing risks of relapse and non‐relapse mortality (NRM). However, the prognostic impact of the LSC17 score on outcomes after allogeneic HCT has not yet been evaluated, particularly for patient cohorts treated more recently given on‐going improvements in HCT outcomes.

Thus, in the present study, we analysed the impact of LSC17 score at diagnosis on outcomes following allogeneic HCT with respect to OS, LFS, RI and NRM. In addition, we evaluated the impact of other transplant parameters including intensity of conditioning and the impact of graft‐versus‐host‐disease (GVHD) development in the context of LSC17 scores.

## 2 METHODS AND PATIENTS

### 2.1 Patient cohorts

**FIGURE 1:**
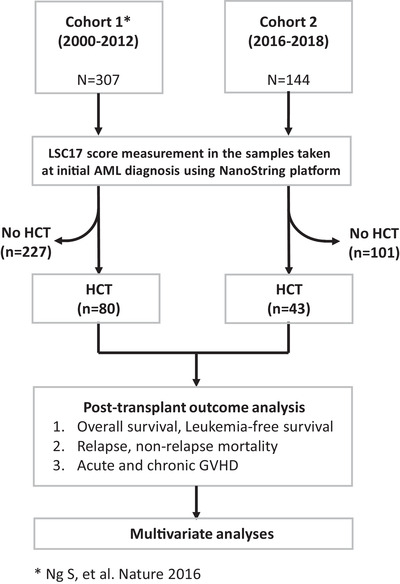
Overview of the study cohorts and analysis

The present study consists of 123 patients comprising two cohorts (Figure 1). Cohort 1 consists of 80 patients who received allogeneic HCT out of 307 patients diagnosed with AML between 2000 and 2012 and were reported previously [11]. Cohort 2 consists of 43 patients who received allogeneic HCT out of 144 AML patients who were prospectively accrued between 2016 and 2018. For both cohorts, LSC17 score was measured using a clinical NanoString assay in diagnostic bone marrow samples, which were stored at the Princess Margaret Leukaemia Tissue Bank, and laboratory staff performing the assay were blinded from patient data. A total of 123 patients were enrolled into the final analysis. The study was approved by the Research Ethics Board at the University Health Network, Toronto, Canada. Transplantation procedures and post‐transplantation management adhered to institutional policy as previously described [3, 12–16].

For the LSC17 score measurement, in brief, marrow samples were processed for RNA extraction. Gene expression profiling was performed using a lab‐validated targeted RNA expression assay on the NanoString nCounter platform (NanoString, Seattle, WA, USA) at the Princess Margaret Advanced Molecular Diagnostics Lab. The LSC17 score was calculated according to an algorithm based on our previous work [11]. The patients were classified as having a high (above median) or low (below median) LSC17 score based on the median LSC17 score of a reference cohort [11].

### 2.2 Patient characteristics and treatment

The patients’ clinical and disease characteristics are summarized in Table 1: sex, male versus female (n = 63 vs. 60; 51.2% vs. 48.8%); age, median of 50 years (range: 18–73); age 60 years or above (n = 33, 26.8%); CR status prior to HCT, CR1 (n = 93, 75.6%), CR2 (n = 30, 24.4%); cytogenetic risk groups [5], favourable (n = 9, 7.3%), intermediate (n = 70, 56.9%), adverse (n = 27, 22.0%), inconclusive or not done (n = 17, 13.8%).

**TABLE 1:** Summary of patient and disease characteristics and transplant procedures with comparison of LSC17 score at AML diagnosis (by Mann–Whitney U test)

Variables	Overall (n = 123)	No. of pts (%)	LSC17 score (median, SE of mean)	*p*‐value
Age at HCT	Age ≥ 60 years	33 (26.8)	0.543 ± 0.041	0.111
	Age < 60 years	90 (73.2)	0.455 ± 0.032	
Sex, male	Male	63 (51.2)	0.467 ± 0.038	0.670
	Female	60 (48.8)	0.490 ± 0.037	
Performance status at diagnosis	KPS 70‐80%	28 (26.9)	0.455 ± 0.052	0.437
	KPS 90‐100%	76 (73.1)	0.513 ± 0.036	
Cytogenetic group by the MRC	Favourable risk	9 (7.3)	0.164 ± 0.088	0.003*
	Intermediate risk	70 (56.9)	0.456 ± 0.032	
	Adverse risk	27 (22.0)	0.618 ± 0.058	
	Inconclusive/not done	17 (13.8)	0.513 ± 0.265	
Disease status at HCT	CR2 or beyond	30 (24.4)	0.397 ± 0.052	0.073
	CR1	93 (75.6)	0.506 ± 0.031	
Conditioning regimen	Reduced intensity	59 (48.0)	0.563 ± 0.034	0.001
	Myeloablative	64 (52.0)	0.401 ± 0.037	
Donor type	Alternative/haploidentical donor	19 (15.4)	0.531 ± 0.071	0.609
	Matched related/unrelated donor	104 (84.6)	0.469 ± 0.028	
Stem cell source	PBSC	115 (93.5)	0.423 ± 0.027	0.454
	BM	8 (6.5)	0.515 ± 0.084	
T‐cell depletion	T‐cell depletion	61 (49.6)	0.533 ± 0.033	0.037
	No T‐cell depletion	62 (50.4)	0.425 ± 0.040	

* *p* = 0.005 between adverse risk (n = 27) vs. others (n = 96, 0.439 ± 0.028).

** Abbreviations: LSC17 score, 17‐gene stemness score; HCT, haematopoietic cell transplantation; KPS, Karnofsky performance status score; MRC, medical research council; CR1, first remission; CR2, second remission; PBSC, peripheral blood stem cells; BM, bone marrow.

The transplant procedure is also summarized in Table 1. A total of 104 patients (84.6%) received grafts from HLA‐matched related or unrelated donors, while 19 (15.4%) received grafts from alternative donors including, mismatched unrelated or haploidentical related donors; 64 patients (52.0%) received a myeloablative conditioning (MAC) regimen and 59 received reduced‐intensity conditioning (RIC) (48.0%). Sixty‐one patients (49.6%) received in vivo T‐cell depletion as GVHD prophylaxis.

### 2.3 Definition of clinical endpoints

The day of the stem cell infusion was defined as day 0. OS was defined as time from the day of HCT until death from any cause or last follow‐up. LFS was defined as time from the day of HCT until relapse or progression of primary disease, or death from any cause or last follow‐up. NRM was defined as mortality following the day of HCT in patients without recurrence of disease. Relapse was defined as the recurrence of primary disease following the HCT. Acute (aGVHD) and chronic GVHD (cGVHD) were diagnosed and graded using the aGVHD consensus conference criteria and the NIH consensus criteria for cGVHD, respectively [17, 18].

### 2.4 Statistical analysis

Primary outcomes were OS and LFS at 2 years after HCT, while relapse incidence (RI) and NRM were evaluated as secondary endpoints. Kaplan–Meier estimates were used for OS and LFS, while the cumulative incidence method was used for RI or NRM considering competing risks. Univariate and multivariate analyses were conducted for OS and LFS using Cox's proportional hazards model and for NRM and RI using the Fine‐Gray model. Outcomes were evaluated according to the LSC17 score group as well as to other clinical risk factors including chronic GVHD as a time‐dependent covariate. Mantel–Byar test and Simon–Makuch plotting were also applied to the statistical analysis [19, 20, 21, 22, 23]. Post‐transplant outcomes, including acute or chronic GVHD development, were analysed and compared according to the LSC17 score groups

Cox proportional hazards regression models for OS and LFS were fit by forcing the LSC17 score groups into all models and using a backward step‐wise selection procedure to identify additional variables from the following: chronic GVHD as a time‐dependent covariate, cytogenetic risk (adverse vs. favourable/intermediate/inconclusive), CR status (CR2 or beyond vs. CR1), donor type (mismatched/haplo donor vs. matched), conditioning intensity (reduced intensity vs. myeloablatve), T‐cell depletion, and age (≥60 years vs. <60 years). Fine‐Grey models for RI and RNM were fit by forcing the LSC17 score group and the same variables were included except for chronic GVHD as a non‐time dependent covariate. Additional analysis was performed for OS, LFS, RI and NRM, including all the pre‐transplant variables, but excluding the covariate of chronic GVHD. Hazard ratios (HR) and 95% confidence intervals (CI) were estimated using a pre‐determined reference risk of 1.0. *p*‐values <0.05 were considered statistically significant. Statistical analyses were performed using EZR software (Saitama Medical Center, Jichi Medical University, Saitama, Japan). EZR (version 1.41) is a modified version of R Commander (version 2.6‐1) (http://www.jichi.ac.jp/saitama‐sct/SaitamaHP.files/statmedEN.html) [24].

## 3 RESULTS

### 3.1 High LSC17 score is associated with adverse risk features

The mean LSC17 score at the time of initial diagnosis in the whole cohort (n = 123) was 0.478 ± 0.026 (range, −0.34 to 1.16, Supporting information Figure S1). The LSC17 score was not different (*p* = 0.491) between patients who received HCT (n = 123; 0.479 ± 0.293 [mean ± S.D.]) and those who did not (n = 227; 0.456 ± 0.295, Supporting information Figure S1).

Among patients who underwent HCT, there was no difference in LSC17 score as a continuous variable according to sex, performance status or donor type (Table 1). A total of 123 patients were allocated to low (n = 65; 52.9%) or high LSC17 score groups (n = 58; 47.1%) based on the median value of LSC17 score in a reference AML cohort [11] (Table 2). Patients with adverse cytogenetic risk made up a higher proportion of the high LSC17 score group (31.0%) compared to the low LSC17 score group (13.8%; *p* = 0.003, Supporting information Table S1). The low LSC17 score group showed a higher proportion of patients who received MAC (60.0%) compared to the high LSC17 score group (43.1%, *p* = 0.061); thus, conditioning regimen was included as a covariate in the multivariate anlaysis. There was a trend to a higher proportion of patients in CR2 or beyond in the low LSC17 score group (31.3%) compared to the high LSC17 score group (17.2%, *p* = 0.081), which is consistent with HCT deferral in lower‐risk patients on attainment of CR1. There was also a trend toward higher LSC17 score in the sub‐group of patients 60 years of age and older (*p* = 0.111). Patients who received RIC had a higher LSC17 score, consistent with the more frequent use of RIC in an older age group (*p* = 0.001).

**TABLE 2:** Comparison of patient and disease characteristics according to LSC17 score group

Category	Group	Overall	Low LSC17 score	High LSC17 score	p‐value
	No of pts (%)	N = 123	N = 65 (52.8)	N = 58 (47.2)	
Age at HCT	years, median (range)	50 (18‐73)	46 (24‐71)	54 (18‐73)	0.209
Age ≥ 60 years		33 (26.8)	16 (24.6)	17 (29.3)	0.557
Sex	female/male	60/63 (48.8/51.2)	29/36 (44.6/55.4)	31/27 (53.4/46.6)	0.328
Performance status at diagnosis* (available in 104 pts)	KPS 90‐100%	76 (73.1)	40 (71.4)	36 (75.0)	0.682
	KPS 70‐80%	28 (26.9)	16 (28.6)	12 (25.0)	
Cytogenetic group by the MRC	Favourable risk	9 (7.3)	9 (13.8)	0 (0)	0.003*
	Intermediate risk	70 (56.9)	40 (61.5)	30 (51.7)	
	Adverse risk	27 (22.0)	9 (13.8)	18 (31.0)	
	Not done/inconclusive	17 (13.8)	7 (10.8)	10 (17.2)	
Disease status at HCT	CR1	93 (75.6)	45 (69.2)	48 (82.8)	0.081
	CR2 or beyond	30 (24.4)	20 (31.3)	10 (17.2)	
Conditioning regimen	Reduced intensity	59 (48.0)	26 (40.0)	33 (56.9)	0.061
	Myeloablative	64 (52.0)	39 (60.0)	25 (43.1)	
Donor type	Matched related	54 (43.9)	26 (40.0)	28 (48.3)	0.647
	Matched unrelated	50 (40.7)	28 (43.1)	22 (37.9)	
	Alternative/haploidentical	19 (15.4)	11 (16.9)	8 (13.8)	
Source of stem cells	PBSC	115 (93.5)	63 (96.9)	52 (89.7)	0.147
	BM	8 (6.5)	2 (3.1)	6 (10.3)	
T‐cell depletion	T‐cell depleted	61 (49.6)	30 (46.2)	31 (53.4)	0.419

* *p* = 0.022 when compared between the adverse cytogenetic group vs. others.

** Abbreviations: BM, bone marrow. council; CR1, first remission; CR2, second remission; HCT, haematopoietic cell transplantation; KPS, Karnofsky performance status score; LSC17 score, 17‐gene stemness core; MRC, medical research; PBSC, peripheral blood stem cells.

### 3.2 Transplant outcomes following allogeneic HCT according to LSC17 score group

With a median follow‐up duration of 46.9 months among survivors after HCT (range 11–227 months), 28 patients experienced relapse (22.8%) while 67 deaths (54.5%) were noted from either relapse (n = 24; 19.5%) or non‐relapse mortality (n = 43; 34.9%). The long‐term outcomes in the 123 patients are as follows: 2‐year OS 52.2% (95% CI 42.5–60.7%), LFS 45.2% (35.7–54.2%), RI 22.9% (15.5–31.1%) and NRM 31.9% (23.6–40.5%).

**FIGURE 2:**
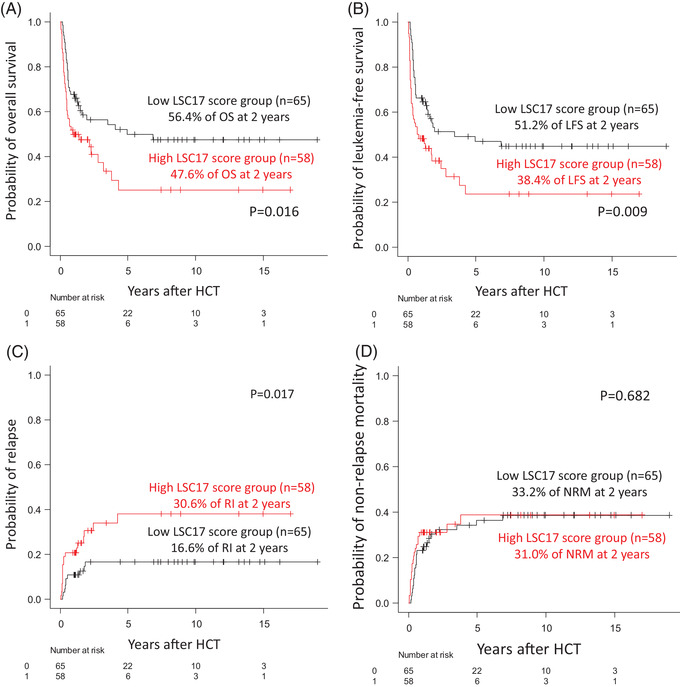
Transplant outcomes in overall patients according to LSC17 score

Transplant outcomes after allogeneic HCT were analysed according to the LSC17 score group (Figure 2). Patients with a low LSC17 score had a better 2‐year OS (56.4%; Figure 2A) and LFS rate (51.2%; Figure 2B) compared to those with a high LSC17 score (47.6%, *p* = 0.016 for OS and 38.4%, *p* = 0.009 for LFS). In addition, there was a significant difference in the 2‐year RI rate (Figure 2C) in favour of the low LSC17 score group (16.6% vs. 30.6% in the high score group, *p *= 0.017). However, there was no difference in NRM at 2 years between the two groups (33.2 vs. 31.0% in the high LSC17 score groups, respectively, *p* = 0.682; Figure 2D).

**FIGURE 3:**
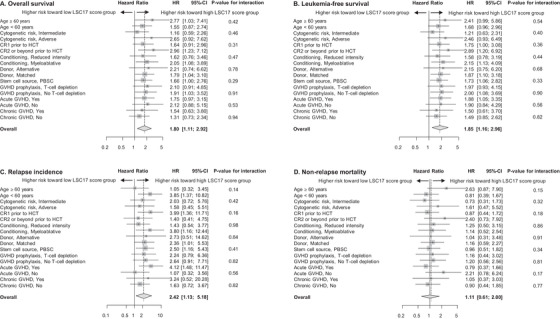
Sub‐group analysis of overall survival (A), leukaemia free‐survival (B), relapse incidence (C) and non‐relapse mortality (D). Hazard ratio and 95% confidence interval are shown for the high LSC17 score group across sub‐groups stratified by age, MRC cytogenetic risk group, complete remission status at HCT, conditioning regimen, stem cell source or T‐cell depletion

Reduced survival and a higher risk of relapse were consistently observed in the high LSC17 score group in comparison to the low LSC17 score group: OS HR 1.80 (1.11–2.92), *p* = 0.018; LFS HR 1.85 (1.16–2.96), *p* = 0.010; and RI HR 2.42 (1.13–5.18), *p* = 0.024. This trend of increased HR in the high LSC17 score group for OS, LFS and RI was consistently observed across all sub‐groups stratified by clinical factors (Figure 3 and Supporting information Table S2). In contrast, the risk of NRM was not significantly different between the two LSC17 score groups (HR: 1.11 [0.61–2.00], *p* = 0.74) as a whole or within sub‐groups (Figure 3 and Supporting information Table S2).

In multivariate analysis, LSC17 score group was confirmed as an independent prognostic factor for both OS and LFS (Table 3). Patients with a high LSC17 score had a twofold higher risk of mortality compared to those in the low LSC17 score group for OS (HR 1.933 [1.185–3.153], *p* = 0.008; Table 3A) as well as LFS (HR 2.009 [1.228–3.286], *p* = 0.002; Table 3B). There was a trend to an increased risk of relapse in the high LSC17 score group (HR 2.145 [0.914–5.033], *p* = 0.079; Table 3C). When pre‐transplant covariates were included in the final statistical model while excluding chronic GVHD, a high LSC17 score was identified as an independent prognostic factor for relapse (HR 2.621 [1.246–5.513, *p* = 0.011; Table 3C). The LSC17 score was not associated with the risk of NRM (*p* = 0.970; Table 3, 3). Overall, these results demonstrate that the LSC17 score measured at AML diagnosis retains prognostic value even after allogeneic HCT in AML patients who subsequently undergo HCT.

**TABLE 3:** Univariate and multivariate analysis of prognostic factors for overall survival (OS), leukaemia‐free survival (LFS), relapse incidence (RI) and non‐relapse mortality (NRM) following allogeneic HCT

A. Overall survival					
		Univariate analysis		Multivariate with step‐wise selection	
Overall survival	Prognostic factor	*p*‐value	HR [95% CI]	*p*‐value	HR [95% CI]
LSC17 score	High LSC17 score group (vs. low)	0.018	1.797 [1.106–2.919]	0.008*	1.933 [1.185–3.153]
Chronic GVHD	Time‐dependent	0.013	0.427 [0.218–0.835]	0.002	0.327 [0.162–0.661]
Cytogenetic risk	Adverse risk (vs. intermediate/favourable)	0.015	1.925 [1.138–3.257]	–	
CR status	CR2 or beyond (vs. CR1)	0.293	1.321 [0.786–2.221]	–	
Donor type	Mismatched/haplo donor (vs. matched)	0.081	1.692 [0.936–3.058]	0.002*	2.825 [1.448–5.513]
Conditioning	Reduced intensity (vs. myeloablative)	0.669	0.898 [0.550‐1.468]	–	
T‐cell depletion	T‐cell depletion (vs. No)	0.106	0.662 [0.401–1.091]	0.001*	0.380 [0.211–0.683]
Age	≥ 60 yrs (vs. < 60 yrs)	0.289	1.340 [0.779–2.301]	–	

B. Leukaemia‐free survival					
		Univariate analysis		Multivariate with step‐wise selection	
Leukaemia‐free survival	Prognostic factor	*p*‐value	HR [95% CI]	*p*‐value	HR [95% CI]
LSC17 score	High LSC17 score group (vs. low)	0.010	1.851 [1.157–2.962]	0.002**	2.009 [1.228–3.286]
Chronic GVHD	Time‐dependent	0.020	0.433 [0.214–0.877]	0.003	0.328 [0.156–0.689]
Cytogenetic risk	Adverse risk (vs. intermediate/favourable)	0.003	2.125 [1.282‐3.521]	–	
CR status	CR2 or beyond (vs. CR1)	0.292	1.317 [0.789–2.198]	–	
Donor type	Mismatched/haplo donor (vs. matched)	0.067	1.705 [0.962‐3.024]	0.003**	2.037 [1.266–3.277]
Conditioning	Reduced intensity (vs. myeloablative)	0.892	1.033 [0.645–1.656]	–	
T‐cell depletion	T‐cell depletion (vs. No)	0.293	0.773 [0.479–1.249]	0.007**	0.473 [0.274–0.818]
Age	≥ 60 yrs (vs. < 60 yrs)	0.140	1.469 [0.881‐2.448]	–	

C. Relapse incidence					
		Univariate analysis		Multivariate with stepwise selection	
Relapse	Prognostic factor	*p*‐value	HR [95% CI]	*p*‐value	HR [95% CI]
LSC17 score	High LSC17 score group (vs. low)	0.024	2.415 [1.126–5.181]	0.079	2.145 [0.914‐5.033]
Chronic GVHD	Development of chronic GVHD (vs. no)	<0.001	0.165 [0.066–0.414]	0.001	0.223 [0.091–0.545]
Cytogenetic risk	Adverse risk (vs. intermediate/favourable)	0.014	2.535 [1.207–5.324]	–	
CR status	CR2 or beyond (vs. CR1)	0.2	1.646 [0.774–3.500]	0.021	2.643 [1.135‐6.156]
Donor type	Mismatched/haplo donor (vs. matched)	0.810	1.124 [0.429–2.938]	–	
Conditioning	Reduced intensity (vs. myeloablative)	0.170	1.690 [0.798–3.575]	–	
T‐cell depletion	T‐cell depletion (vs. No)	0.690	1.165 [0.549–2.469]	–	
Age	≥ 60 yrs (vs. < 60 yrs)	0.170	1.695 [0.794–3.617]	–	

(Continues)

**TABLE 3:** (Continued)

D. Non‐relapse mortality					
		Univariate analysis		Multivariate with step‐wise selection	
Non‐relapse mortality	Prognostic factor	*p*‐value	HR [95% CI]	*p*‐value	HR [95% CI]
LSC17 score	High LSC17 score group (vs. low)	0.740	1.108 [0.612–2.005]	0.970	0.987 [0.524–1.860]
Chronic GVHD	Development of chronic GVHD (vs. no)	<0.001	2.688 [1.495–4.831]	<0.001	2.959 [1.590–5.495]
Cytogenetic risk	Adverse risk (vs. intermediate/favourable)	0.470	1.287 [0.645–2.568]	–	
CR status	CR2 or beyond (vs. CR1)	0.950	1.022 [0.507–2.059]	–	
Donor type	Mismatched/haplo donor (vs. matched)	0.1	1.796 [0.886–3.639]	0.013	2.759 [1.235–6.164]
Conditioning	Reduced intensity (vs. myeloablative)	0.250	0.698 [0.378–1.290]	–	
T‐cell depletion	T‐cell depletion (vs. No)	0.110	0.605 [0.326–1.123]	0.010	0.375 [0.177–0.791]
Age	≥ 60 yrs (vs. < 60 yrs)	0.730	1.119 [0.586–2.137]	–	

Abbreviations: 95% CI, 95% confidence interval; CR2, second remission.; GVHD, graft‐versus‐host disease; HR, hazard ratio; LSC17 score, 17‐gene stemness core.

* LSC17 score (*p* = 0.008, HR 1.941 [1.186–3.175), donor type (*p* = 0.006, HR 2.511 [1.306–4.828] and T‐cell depletion (*p* = 0.015, HR 0.503 [0.289–0.875]) when analysed in multivariate analysis with step‐wise selection procedure including all the pre‐transplant variables above but excluding chronic GVHD.

** LSC17 score (*p* = 0.005, HR 2.009 [1.228–3.286]), donor type (*p* = 0.005, HR 2.511 [1.306–4.828] and T‐cell depletion (*p* = 0.015, HR 0.503 (0.289–0.875]) were significantly associated with leukaemia‐free survival when analysed in multivariate analysis with step‐wise selection procedure including all the pre‐transplant variables above but excluding chronic GVHD.

*** LSC17 score (*p* = 0.011, HR 2.621 [1.246–5.513]) and CR status (*p* = 0.037, HR 1.901 [1.082‐4.009]) were significantly associated with relapse risk when analysed in multivariate analysis with step‐wise selection procedure including all the pre‐transplant variables above but excluding chronic GVHD.

**** Donor type (*p* = 0.017, HR 2.811 [1.203‐6.569] and T‐cell depletion (*p* = 0.022, HR 0.426 [0.205–0.886]) were identified as independent prognostic factors for NRM, while the LSC17 score was not significant (*p* = 0.490, HR 1.237 [0.677–2.257]) when multivariate analysis was analysed including all the pre‐transplant variables above but excluding chronic GVHD and LSC17 score was forced in the final model.

### 3.3 Impacts of conditioning regimen intensity on outcomes in high and low LSC score groups

To determine whether a more intensive conditioning regimen (i.e., MAC can reduce the risk of relapse and improve transplant outcomes in patients with a high or low LSC17 score, we analysed the impact of conditioning regimen type (MAC vs. RIC) on outcomes separately in both sub‐groups. As noted above, the high LSC17 score group received RIC more frequently than those with low LSC17 score, possibly due to the association of higher LSC score with older age and more frequent use of RIC in older patients.

Within both low and high LSC17 score groups, there were no significant differences in OS, LFS and NRM between patients who received MAC or RIC (Supporting information Figure S2). There was a trend toward increased RI at 2 years in patients who received RIC compared to MAC in the low LSC17 score group (10.3% in RIC vs. 30.4% in MAC; *p* = 0.093), while in the high LSC17 score group the incidence of relapse at 2 years was very similar between the MAC and RIC groups: 25.7 vs. 35.0% (*p* = 0.943). These results suggest that augmentation of conditioning intensity may not improve clinical outcome, particularly for those with a high LSC17 score, although the relatively small sample size precludes drawing any firm conclusions.

### 3.4 Development of chronic GVHD is associated with better outcomes, following HCT in high and low LSC score groups

Acute GVHD was observed in 74 of 123 patients (60.2%) with 55.3% (46.0–63.6%) incidence at day 120 and the following distribution according to grade: grade 1 (n = 17, 13.8%), grade 2 (n = 34, 27.6%), grade 3 (n = 20, 16.3%) and grade 4 (n = 6, 4.9%). There were no differences in acute GVHD incidence between LSC17 score groups (*p* = 0.389), or in the distribution of cases by acute GVHD grade. Development of acute GVHD as a time‐dependent covariate was associated with adverse OS (Supporting information Figure S4) and LFS (Supporting information Figure S5).

A total of 61 patients (49.6%) developed chronic GVHD, with mild (n = 29, 23.6%), moderate (n = 26, 21.1%) and severe grades (n = 6, 4.9%) at initial presentation of chronic GVHD by the NIH consensus criteria global scoring system. Higher grade cGVHD tended to occur more frequently in the low LSC17 score group (*p* = 0.065), with an incidence of chronic GVHD of 48.2% (38.8–57.0%) at 2 years. Of note, there was a significantly lower incidence of chronic GVHD in the high LSC17 score group compared to the low score group (*p* = 0.019). However, in a multivariate analysis for chronic GVHD risk factors, the LSC17 score group was not found to be an independent risk factor (*p* = 0.096 when analysed together with other confounding variables such as conditioning intensity, donor type, stem cell source and T‐cell depletion; Supporting information Table S3).

**FIGURE 4:**
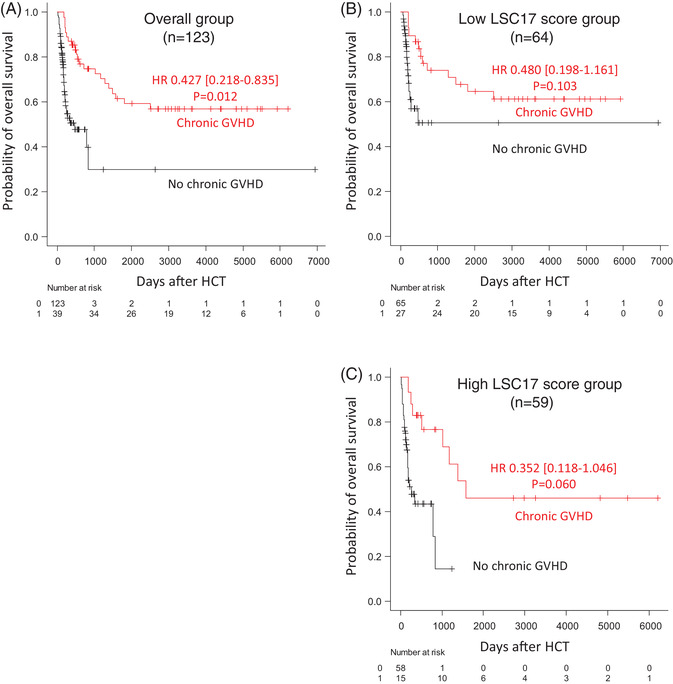
Simon–Makuch plot of overall survival considering time‐dependent variable of chronic GVHD in overall patients (A), in the low LSC17 score group (B) and in the high LSC17 score group (C), suggesting favourable effect of chronic GVHD on overall survival

The development of chronic GVHD has been shown to protect patients from relapse, thus, favourably impacting OS, LFS and RI [25, 26, 27]. In multivariate analysis (Table 3), chronic GVHD was associated with better OS and LFS (as primary end‐points) when considered a time‐dependent covariate, analysed by the Mantel–Byar test [19, 20, 21, 22, 23]. In the entire cohort of patients who underwent HCT, OS was significantly better in patients who developed chronic GVHD (*p* = 0.012; Figure 4A). Within both LSC17 score groups, we observed the same trend of benefit from chronic GVHD on OS in both the low (*p* = 0.103; Figure 4B) and high score groups (*p* = 0.06; Figure 4C). Mantel–Byar analysis of LFS showed a similar pattern of results in the overall (*p* = 0.02) and low LSC17 score groups (p = 0.05) (Supporting information Figure S3). These results strongly suggest that modulation of the graft‐versus‐leukaemia (GVL) effect mediated by chronic GVHD could be utilized to overcome adverse disease features after HCT in patients with a high LSC17 score.

## 4 DISCUSSION

Intrinsic leukaemiastem cell properties (LSCs) are associated with treatment resistance and relapse risk in AML. The LSC17 score measured at diagnosis robustly predicts outcomes and treatment response in AML patients treated with standard induction chemotherapy [11]. Patients with a high LSC17 score had poor outcomes with current treatments including allogeneic HCT. In our previous study [11], we evaluated whether allogeneic HCT could improve OS when considering HCT as a time‐dependent covariate compared to the non‐HCT approach. We concluded that HCT did not benefit both sub‐groups of high (*p* = 0.2) or low (*p* = 0.06) LSC17 score patients, possibly due to the competing risks of relapse and NRM. In the current study, we carried out a more in‐depth analysis of transplant‐related outcomes, and examined the impact of conditioning regimen intensity and development of GVHD in the context of LSC17 scores in a large cohort of AML patients who underwent HCT.

Our results show that the LSC17 score measured at the time of AML diagnosis retains prognostic impact in patients who undergo allogeneic HCT. A high LSC17 score was associated with a higher risk of relapse and worse LFS after HCT, thus, affecting OS adversely. The LSC17 score retained independent prognostic value for OS and LFS in multivariate analysis. A more intensive conditioning regimen (i.e., MAC) did not improve clinical outcomes after HCT, particularly in the high LSC17 score patients. Together, these results demonstrate that despite the achievement of CR, the adverse biology and stemness features reflected by a high LSC17 score may only be partially overcome by HCT, highlighting an urgent need for novel HCT therapeutic strategies. The LSC17 score is a powerful tool for identifying patients prior to HCT who are at high risk of relapse and poor transplant‐related outcomes, for possible enrolment in clinical trials evaluating new treatment approaches for this low‐risk group of patients.

Interestingly, the development of chronic GVHD reduced the risk of relapse and improved transplant outcomes in both LSC17 score groups. GVHD runs in parallel with GVL effect [25, 26, 27], and our results suggest that chronic GVHD may provide a beneficial effect that helps to mitigate the adverse prognosis conferred by a high LSC17 score. Thus, the induction of a GVL effect could be a useful strategy to improve outcomes in the management of high‐risk patients. The LSC17 score could be an informative biomarker to incorporate into clinical decisions regarding GVHD prophylaxis, including T‐cell depletion, although more study is certainly required. Additionally, tapering of systemic immunosuppressive therapy could be accelerated, or prophylactic donor lymphocyte infusions can be considered in patients with a high LSC17 score to induce a GVL effect, should no chronic GVHD be observed following HCT.

The current cohort consists of two different sets of patients, one treated between 2000 and 2012 who were retrospectively accrued into the study, while the other was enrolled prospectively between 2016 and 2018. The LSC17 score was measured by the same clinical assay in all patients, and there was no difference in the distribution of LSC17 scores between patients who received HCT and those who did not. We expected that more patients with high LSC17 score would have received HCT, as HCT is usually deferred in patents with favourable cytogenetic risk until the time of relapse. However, we did not detect any difference in the proportion of high and low LSC17 scores in patients that received HCT compared to those that did not. Although HCT is indicated for patients with high‐risk disease features, such as adverse cytogenetics, patients with high LSC17 scores are less likely to achieve CR [11] and, thus, may be less likely to proceed to HCT.

In this study, patients were classified as having a high or low LSC17 score based on the median score of a previously published reference cohort [11]. This score was originally trained on OS outcomes in a large, diverse AML cohort and validated in several independent cohorts by our group and others [28]. It is possible that the cut‐off between high and low scores could be optimized for cohorts enriched for AML patients who have undergone HCT to improve prognostic stratification power in this group of patients. Along these lines, the LSC17 score was recently shown to be prognostic in paediatric AML, but with an optimal 25:75 split [29, 30]. A sub‐score of 4 of the 17 LSC genes was recently reported to be prognostic in MDS [31]. This and other previously reported leukaemia gene expression signatures [32, 33] could be examined in future studies to determine their predictive or prognostic value in the setting of allogeneic HCT in AML patients.

Other limitations of this study include its retrospective nature, and the relatively small cohort with some heterogeneity. In addition, the observed outcomes from the current study have not been validated in an independent cohort. A further limitation is that only those patients reaching and undergoing HCT have been included, and thus, cannot replace an intention‐to‐treat prospective study. The LSC17 Nanostring assay is undergoing development with evaluation in an on‐going prospective study, and remains, at present, a research tool.

In conclusion, the LSC17 score is highly prognostic for mortality and relapse risk following allogeneic HCT. Accordingly, it would be useful to incorporate the LSC17 score for risk stratification of AML patients undergoing HCT to inform clinical decision‐making for GVHD prophylaxis and post‐transplant management. Patients with a high LSC17 score should be enrolled in clinical trials to evaluate novel therapeutic strategies or refinement of current therapies to reduce the risk of relapse and improve outcomes after allogeneic HCT.

## CONFLICT OF INTEREST

The authors declare they have no conflicts of interest.

## ETHICS STATEMENT

This study was approved by the Institutional Ethics Review Board at University Health Network and was conducted following the Declaration of Helsinki.

## Supporting information

Supporting Information

Supporting Information
