# Supplementary material for: The 17‐gene stemness score associates with relapse risk and long‐term outcomes following allogeneic haematopoietic cell transplantation in acute myeloid leukaemia
Source: EJHaem. 2022 May 23;3(3):873–84. doi: 10.1002/jha2.466 (PMC9422016; doi:10.1002/jha2.466)
Supplement: Supplementary file 1 — Supporting Information [file JHA2-3-873-s001.docx]

**Supplementary Figures.**

**Supplementary Figure 1. Distribution of LSC17 score in patients who received allogeneic hematopoietic stem cell transplantation (HCT; A), and comparison of the LSC17 score between patients who did or did not receive HCT (B).**

**Supplementary Figure 2. Subgroup analysis according to the conditioning regimen intensity in low LSC17 score (A-D) and high LSC17 score (E-H) groups**

**Supplementary Figure 3. Simon-Makuch plot of the impact of time-dependent occurrence of chronic GVHD on leukemia-free survival in overall patients (A), in the low LSC17 score group (B) and in the high LSC17 score group (C), suggesting a favorable effect of chronic GVHD on leukemia-free survival.**

**Supplementary Figure 4. Simon-Makuch plot of the impact of time-dependent occurrence of acute GVHD on OS in overall patients (A), in the low LSC17 score group (B) and in the high LSC17 score group (C) suggesting an adverse effect of acute GVHD on leukemia-free survival.**

**Supplementary Figure 5. Simon-Makuch plot of the impact of time-dependent occurrence of acute GVHD on leukemia-free survival in overall patients (A), in the low LSC17 score group (B) and in the high LSC17 score group (C), suggesting an adverse effect of acute GVHD on leukemia-free survival.**

**Supplementary Figure 1. Distribution of LSC17 score in patients who received allogeneic hematopoietic stem cell transplantation (HCT; A), and comparison of the LSC17 score between patients who did or did not receive HCT (B).**

|  |  |
| --- | --- |

**Supplementary Figure 2. Subgroup analysis according to the conditioning regimen intensity in low LSC17 score (A-D) and high LSC17 score (E-H) groups**

|  |  |
| --- | --- |
|  |  |
|  |  |
| *Abbreviations: RIC, reduced intensity; MAC, myeloablative conditioning | |
|  |  |
|  |  |

**Supplementary Figure 3. Simon-Makuch plot of the impact of time-dependent occurrence of chronic GVHD on leukemia-free survival in overall patients (A), in the low LSC17 score group (B) and in the high LSC17 score group (C), suggesting a favorable effect of chronic GVHD on leukemia-free survival.**

|  |  |
| --- | --- |
|  |  |

**Supplementary Figure 4. Simon-Makuch plot of the impact of time-dependent occurrence of acute GVHD on OS in overall patients (A), in the low LSC17 score group (B) and in the high LSC17 score group (C)** **suggesting an adverse effect of acute GVHD on leukemia-free survival.**

|  |  |
| --- | --- |
|  |  |

**Supplementary Figure 5. Simon-Makuch plot of the impact of time-dependent occurrence of acute GVHD on leukemia-free survival in overall patients (A), in the low LSC17 score group (B) and in the high LSC17 score group (C), suggesting an adverse effect of acute GVHD on leukemia-free survival.**

|  |  |
| --- | --- |
|  |  |
