# Supplementary material for: The 17‐gene stemness score associates with relapse risk and long‐term outcomes following allogeneic haematopoietic cell transplantation in acute myeloid leukaemia
Source: EJHaem. 2022 May 23;3(3):873–84. doi: 10.1002/jha2.466 (PMC9422016; doi:10.1002/jha2.466)
Supplement: Supplementary file 2 — Supporting Information [file JHA2-3-873-s002.docx]

**Supplementary Table 1. Summary of disease characteristics and transplant procedures according to LSC17 score group**

| **Category** | **Group** | **Overall** | **Low LSC17 score** | **High LSC17 score** | **p-value** |
| --- | --- | --- | --- | --- | --- |
|  | No of pts (%) | N=123 | N=65 (52.8) | N=58 (47.2) |  |
| Cytogenetic group by the MRC | Favorable risk | 9 (7.3) | 9 (13.8) | 0 (0) | 0.003* |
|  | Intermediate risk | 70 (56.9) | 40 (61.5) | 30 (51.7) |  |
|  | Adverse risk | 27 (22.0) | 9 (13.8) | 18 (31.0) |  |
|  | Not done/inconclusive | 17 (13.8) | 7 (10.8) | 10 (17.2) |  |
| Conditioning intensity | Reduced intensity | 59 (48.0) | 26 (40.0) | 33 (56.9) | 0.061 |
|  | Myeloablative | 64 (52.0) | 39 (60.0) | 25 (43.1) |  |
| Conditioning regimen† | Bu4Flu4 +/- low dose TBI | 40 (32.5) | 26 (40.0) | 14 (24.1) | 0.353 |
|  | CY-TBI / AraC-CY-TBI | 10/11 (8.1/8.9) | 6/6 (9.2/9.2) | 4/5 (6.9/8.6) |  |
|  | VP16-TBI / Bu4-VP16 | 2/1 (1.6/0.8) | 1/0 (1.5/0) | 1/1 (1.7/1.7) |  |
|  | Bu2Flu4 +/- low dose TBI | 59 (48.0) | 26 (40.0) | 33 (56.9) |  |
| GVHD prophylaxis‡ | CSA/PTCy | 40 (32.5) | 15 (23.1) | 25 (43.1) | 0.092 |
|  | CSA/MMF | 40 (32.5) | 25 (38.5) | 15 (25.9) |  |
|  | CSA/MTX | 28 (22.8) | 15 (23.1) | 13 (22.4) |  |
|  | CSA alone | 15 (12.2) | 10 (15.4) | 5 (8.6) |  |
| T-cell depletion | T-cell depleted | 61 (49.6) | 30 (46.2) | 31 (53.4) | 0.419 |
| Donor type | Matched related | 54 (43.9) | 26 (40.0) | 28 (48.3) | 0.647 |
|  | Matched unrelated | 50 (40.7) | 28 (43.1) | 22 (37.9) |  |
|  | Alternative/haploidentical | 19 (15.4) | 11 (16.9) | 8 (13.8) |  |
| Source of stem cells | PBSC | 115 (93.5) | 63 (96.9) | 52 (89.7) | 0.147 |
|  | BM | 8 (6.5) | 2 (3.1) | 6 (10.3) |  |

*Abbreviations: MRC, Medical Research Council; TBI, total body irradiation; GVHD, graft versus host disease; CSA, cyclosporine; PTCy, post-transplant cyclophosphamide; MMF, mycophenolate mofetil; MTX, methotrexate; PBSC, peripheral blood stem cells; BM, bone marrow.

† Bu4Flu4, fludarabine 50 mg/m^2^ × 4 days, busulfan 3.2 mg/kg × 4 days ± low dose TBI, 2 Gy, 2 fractions; CY-TBI, cyclophosphamide 60mg/kg × 2 days, TBI 2 Gy twice daily × 3 days; AraC-CY-TBI, cytarabine 1400 g/m^2^, cyclophosphamide 60mg/kg × 2 days, TBI 5 Gy single fraction; VP16-TBI etoposide 60mg/kg × 1 day, TBI 2 Gy twice daily × 3 days; Bu4-VP16 etoposide 60mg/kg × 1 day, busulfan 3.2 mg/kg × 4 days; Bu2Flu4 busulfan 3.2 mg/kg × 2 days, fludarabine 30 mg/m^2^ × 4 days ± 2 cGy TBI single fraction.

‡ CSA/PTCy, PTCy, 50 mg/kg IV on days +3 and +4, CSA 2.5 mg/kg IV q12h from D+5; CSA/MMF CSA 2.5 mg/kg IV q12h, MMF 15 mg/kg q8h × 30 days; CSA/MTX, CSA 2.5 mg/kg IV q12h, MTX, 15 mg/m^2^ on day +1 and 10 mg/m^2^ on days +3, +6, and +11; CSA alone CSA 2.5 mg/kg IV q12h from D0

**Supplementary Table 2. Subgroup analysis of multivariate analysis for prognostic factors with respect to the overall survival (OS), leukemia-free survival (LFS), relapse incidence (RI) and non-relapse mortality (NRM) in the group confined to those received myeloablative (n=65) vs. reduced intensity conditioning (n=58), respectively.**

| **Prognostic factor** | **Overall survival** | | **Leukemia-free survival** | | **Relapse incidence** | | **Non-relapse mortality** | |
| --- | --- | --- | --- | --- | --- | --- | --- | --- |
|  | *P* value | HR (95% CI) | *P* value | HR (95% CI) | *P* value | HR (95% CI) | *P* value | HR (95% CI) |
| **Overall (n=123)** | | | | | | | | |
| LSC17 score* | 0.008 | 1.979 [1.193-3.283] | 0.011 | 1.910 [1.160-3.145] | - | | - | |
| Chronic GVHD | 0.009 | 0.401 [0.201-0.800] | 0.018 | 0.413 [0.198-0.863] | <0.001 | 0.165 [0.066-0.414] | <0.001 | 0.372 [0.207-0.669] |
| Disease status, CR2 | 0.043 | 1.764 [1.018-3.056] | 0.024 | 1.891 [1.088-3.286] | - | | - | |
| Adverse cytogenetics | - | | 0.014 | 1.907 [1.137-3.199] | - | | - | |
| Age (≥ 60 yrs) | - | | **-** | | - | | - | |
| **Myeloablative conditioning (n=65)** | | | | | | | | |
| LSC17 score* | 0.017 | 2.207 [1.153-4.226] | 0.017 | 2.190 [1.150–4.172] | 0.017 | 3.985 [1.278–12.43] | - | |
| Chronic GVHD | 0.040 | 0.415 [0.179-0.962] | - | | - | | <0.001 | 0.199 [0.091-0.436] |
| Disease status, CR2 | - | | - | | 0.048 | 3.410 [1.011-11.50] | - | |
| Adverse cytogenetics | - | | 0.043 | 2.246 [1.025–4.925] | - | | - | |
| Age (≥ 60 yrs) | - | | - | | 0.013 | 6.946 [1.516–31.82] | - | |
| **Reduced intensity conditioning (n=58)** | | | | | | | | |
| LSC17 score* | - | | - | | - | | - | |
| Chronic GVHD | 0.020 | 0.229 [0.065-0.797] | 0.014 | 0.204 [0.057-0.725] | <0.001 | 1.3x10^-5^ [7.15x10^-6^-2.4x10^-5^] | - | |
| Disease status, CR2 | - | | - | | - | | - | |
| Adverse cytogenetics | - | | - | | - | | - | |
| Age (≥ 60 yrs) | - | | - | | - | | - | |

* Abbreviations: HR, hazard ratio; 95% CI, 95% confidence interval; LSC17 score, 17-gene stemness core; GVHD, graft-versus-host disease; CR2, second remission

**Supplementary Table 3. Multivariate analysis for the risk factor of acute/chronic GVHD development**

1. **Risk factor analysis for acute GVHD development**

|  |  | **Univariate** | | **Multivariate** | | |
| --- | --- | --- | --- | --- | --- | --- |
| **Risk factor** | **Risk group** | **Incidence at 120 days (%)** | **P-value** | **HR** | **95%CI** | **p-value** |
| LSC17 score group | Low LSC17 score | 60.0 (46.9-70.9) | 0.389 | 1.000 |  | 1.000 |
|  | High LSC17 score | 50.0 (36.4-62.1) |  | 0.998 | 0.615-1.621 |  |
| Conditioning regimen | Myeloablative | 68.8 (55.6-78.7) | 0.002 | 1.000 |  | 0.024 |
|  | Reduced intensity | 40.7 (28.0-52.9) |  | 0.521 | 0.295-0.919 |  |
| Donor type | Matched donor | 56.3 (46.1-65.3) | 0.440 | 1.000 |  | 0.099 |
|  | Alternative donor | 50.0 (26.3-69.8) |  | 1.788 | 0.897-3.564 |  |
| T-cell depletion | No T-cell depletion | 67.7 (54.3-*78.0) | 0.024 | 1.000 |  | 0.230 |
|  | T-cell depletion | 42.6 (30.0-54.7) |  | 0.699 | 0.387-1.262 |  |

1. **Risk factor analysis for chronic GVHD development**

|  |  | **Univariate** | | **Multivariate** | | |
| --- | --- | --- | --- | --- | --- | --- |
| **Risk factor** | **Risk group** | **Incidence at 2 years (%)** | **P-value** | **HR** | **95%CI** | **p-value** |
| LSC17 score group | Low LSC17 score | 58.2 (44.6-69.6) | 0.019 | 1.000 |  | 0.096 |
|  | High LSC17 score | 36.6 (24.1-49.1) |  | 0.616 | 0.348-1.089 |  |
| Conditioning regimen | Myeloablative | 53.8 (40.4-65.5) | 0.103 | 1.000 |  | 0.220 |
|  | Reduced intensity | 40.7 (28.0-53.0) |  | 0.682 | 0.369-1.257 |  |
| Donor type | Matched donor | 49.9 (39.4-59.5) | 0.341 | 1.000 |  | 0.590 |
|  | Alternative donor | 40.0 (17.8-61.5) |  | 0.811 | 0.377-1.746 |  |
| T-cell depletion | No T-cell depletion | 51.6 (38.1-63.5) | 0.341 | 1.000 |  | 0.540 |
|  | T-cell depletion | 43.9 (30.6-56.5) |  | 0.837 | 0.476-1.470 |  |
